# The challenge of opt-outs from NHS data: a small-area perspective

**DOI:** 10.1093/pubmed/fdy059

**Published:** 2018-03-26

**Authors:** Frédéric B Piel, Brandon L Parkes, Hima Daby, Anna L Hansell, Paul Elliott

**Affiliations:** UK Small-Area Health Statistics Unit, MRC-PHE Centre for Environment & Health, School of Public Health, Imperial College London, London, UK

## Introduction

One of the founding principles of the NHS is that it offers comprehensive, universal and free public health services at the point of delivery. As a result, NHS data provide a huge and invaluable resource of routinely collected primary (e.g. visits to GP practices) and secondary (e.g. hospital admissions, outpatient appointments, A&E attendances) healthcare data covering near-100% of the population of England. NHS Digital has the responsibility for collecting and publishing data and information from across the health and social care system in England and controls the dissemination of these data. Detailed analysis of NHS data by public health and research institutions has the potential to considerably improve health and social care in England.

Data privacy and confidentiality of identifiable health data are a legal responsibility of the NHS, and a source of concern for patients. As illustrated by recent controversies and cyber-attacks,^1,2^ ensuring data security has become a growing technological challenge for the NHS, for example, with the move towards electronic health records. As per the NHS Constitution,^3^ patients have the right to request that their confidential information is not used beyond their own care and treatment. This right was formalized in January 2014 through an opt-out system, following a recommendation made by the National Data Guardian (NDG) for Health and Care, Dame Fiona Caldicott, in her 2013 report.^4^ A National Data Opt-out Programme is scheduled to replace the current two types of opt-outs from March 2018, following new recommendations made by the NDG in her Review of data security, consent and opt-outs published in 2016.^5^

Public health research, including disease surveillance, can contribute vital information for the direct care and treatment of patients,^6,7^ as well as broader issues related to population health,^8^ but many studies are no longer granted access to the full national health datasets held by NHS Digital. Our aim here is to comment, ahead of the launch of the new National Data Opt-out Programme, on the potential consequences of these non-random gaps in national health databases. This is particularly a concern for research that is currently unable to access complete health data, i.e. where opt-outs are upheld, such as, for example, small-area health studies of non-communicable diseases.^9^

## Types of opt-outs

The default system in place across the NHS is one of implied consent, so that healthcare professionals can share personal confidential data to provide optimum care. This should be done according to strict NICE guidelines.^10^

**Fig. 1:**
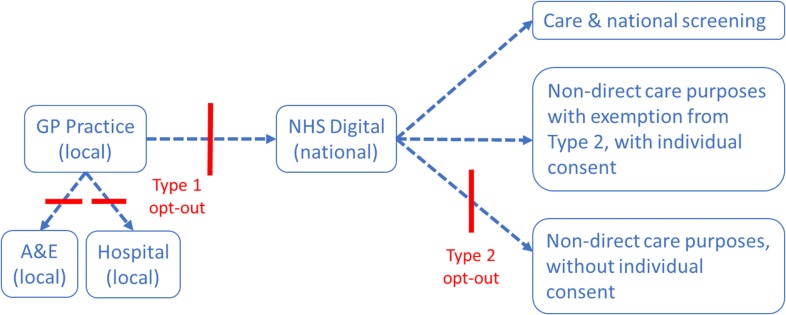
Schematic of the flow of personal confidential health information, and the role of types 1 and 2 opt-outs on data sharing within the NHS and approved data users. Adapted from MRC document on type 2 opt-outs available at www.mrc.ac.uk/documents/pdf/nhs-digital-and-type-2-patient-opt-outs-voct2016.

An opt-out system, including two different types of opt-outs—types 1 and 2—was launched in January 2014, following a recommendation from the 2013 Caldicott report (Fig. 1).^4^ The type 1 opt-out prevents information being shared outside a GP practice for purposes other than direct care. This means that, in case of an emergency, health professionals outside a patient’s practice are not able to view the individual’s health records. Furthermore, unless patients consent to participate in a specific study (e.g. cohort or clinical trial), data relating to type 1 opt-outs cannot be used for research purposes. Type 2 opt-outs, which continue to apply following a patient’s death, prevent information being shared outside of national databases managed by NHS Digital for purposes beyond the individual’s direct care. Type 2 opt-outs do not apply when (i) information is used to support the patient’s direct care and treatment; (ii) there is specific patient consent for a specific purpose (e.g. clinical trial); (iii) there is a mandatory legal requirement (e.g. court order); (iv) the information is not deemed personal confidential information (e.g. release of some demographics information only); (v) information is made available in anonymised form (e.g. aggregated data); and (vi) information is used to support the management of communicable diseases (e.g. infectious disease outbreaks) and other risks to public health. This last exception falls under the legal basis of Regulation 3 of the Health Service (Control of Patient Information) Regulations 2002. A notable absence here relates to a specific mention of the management, including routine surveillance, of non-communicable diseases, such as cardiovascular and respiratory diseases, nor of birth or reproductive effects (e.g. congenital anomalies).

In theory, healthcare professionals should advise patients about the benefits of sharing data and the choices they can make, and where they can find more information which will enable them to make an informed decision regarding the use of their personal confidential data. Opt-out requests should be made explicitly by informed individuals at their local GP practice, not by GPs or other healthcare professionals on behalf of their patients, although for children aged under 16, the duty of care to the child has top priority.

From March 2018, a new National Data Opt-outs Programme will be rolled out to replace the current types 1 and 2 opt-outs across the NHS in England (https://digital.nhs.uk/national-data-opt-out). With the new programme, due to be implemented by March 2020, patients will be able to directly set their national data opt-out preferences online (with a non-digital alternative being offered to those who cannot or do not want to use the online system).

## How frequent are opt-outs?

Statistical publications on types 1 and 2 opt-outs were released online by NHS Digital (http://content.digital.nhs.uk/careinfochoices) on a monthly basis between April and December 2016, and are available on a quarterly basis since January 2017. NHS Digital reported 1 806 882 (3.07%) and 1 403 335 (2.39%) instances of types 1 and 2 opt-outs, respectively, in December 2017. Although in theory, patients requesting a type 2 opt-out should be in addition to those who requested a type 1 opt-out (in which case, a total of 5.46% of NHS patients would have opted out), the relationship between the two types of opt-outs appears to vary between GP practices. Although the exact overall number of patients who opted out, either through type 1 or 2 (or both), cannot be calculated from data released by NHS Digital, we would expect some double-counting between the two types of opt-outs, resulting in a lower national prevalence than the 5.46% given above.

## High-level analysis of type 1 opt-outs at Clinical Commissioning Group level (*n* = 207)

Based on a high-level analysis at the Clinical Commissioning Group (CCG) level of Hospital Episode Statistics (HES) Admitted Patient Care (APC), Outpatients (OP) and Accident & Emergency (A&E) 2015–16 annual datasets conducted by NHS Digital in January 2017,^11^ type 2 opt-outs were more frequent in specific sub-groups of the population. OP data suggest that opt-out rates are significantly higher (*P* < 0.0001) in patients aged in their 60 s (3.67%) and 70 s (3.81%) compared with those in their 20 s (2.48%), in females (3.03%) compared with males (2.68%), and in patients whose ethnicity was recorded as black (4.61%) compared with white (2.87%) or Asian (1.91%). Age, sex, ethnicity and socio-economic status are important confounders in health studies. This analysis also revealed important geographical variations with type 2 opt-out rates ranging between 0.61% (West Yorkshire) and 11.51% (Greater Manchester).

## Detailed analysis of type 2 opt-outs at GP practice level (*n* = 7419)

Type 2 opt-out data at small-area level and rates by age, sex and ethnicity at the GP practice level are not released by NHS Digital. However, analysis of accessible data provides the opportunity to explore temporal trends, spatial patterns and possible data quality issues. In December 2017, out of 6854 GP practices for which an opt-out prevalence was available, 188 had an opt-out prevalence >10%, including 24 with a prevalence >50%.

### Data quality

The quality of data on opt-outs depends on GP practices reporting both the number of opt-outs (numerator) and the overall number of patients registered at GP practices (denominator). Overall, the vast majority of GP practices report both counts.

There were 58,880,877 patients registered at GP practices on 1 December 2017.^12^ This is higher than the overall estimated population of England in mid-2017 (55 765 513)^13^ suggesting that a substantial number of patients might be registered in more than one GP practice or have died but not been removed from registers—often termed ‘ghost patients’.^14^ We have carried out analysis of opt-out data from consecutive time periods and identified a range of inconsistencies which could partly explain the differences in numbers of patients registered, including (i) GP practices reporting relatively implausible decreases (up to 65%) in either the number of patients registered or patients opting out; and (ii) double-counting of patients for several months after the merging of several local GP practices.

A handful of GP practices systematically report more type 2 opt-outs than registered patients (i.e. prevalence >100%). NHS Digital’s explanation for this is that the number of patient opt-outs includes patients other than those currently registered for General Medical Service (GMS), including patients not registered for GMS but registered for another service (e.g. Personal Medical Services and Alternative Provider Medical Services), deceased patients, and patients who have left the practice. Nevertheless, an opt-out prevalence above 90% in a GP practice with thousands of patients—which is the case for all GP practices reporting such a prevalence—is statistically very unlikely. This therefore raises important questions about whether all these patients explicitly requested that they should be opted out.

### Temporal analyses of types 1 and 2 opt-outs

The prevalence of opt-outs has been slowly increasing over time between April 2016 and December 2017 from 2.50 to 3.07% and from 2.17 to 2.39% for types 1 and 2, respectively (Table 1). Their prevalence increased relatively linearly by 0.03% (∼20 000 patients) and 0.01% (∼10 000 patients) per month, respectively over the period studied. There is currently no straightforward mechanism for patients to re-opt-in after they opted out, which poses a long-term problem, particularly in areas with high opt-out rates. The reasons underlying this increase are not clear and further research involving patients from selected areas would be informative. Because opt-outs are influenced by age, sex, ethnicity and socio-economic status, resulting in a non-random distribution, such a trend has important implications for the generalizability of public health research on the impact on health of long-term exposures to environmental risk factors, such as, for example, air pollution^15^ or water contamination.^16^

**Table 1:** Summary of the number of patients registered and patients opting out in England, based on statistical publications released monthly (between April and December 2016) or quarterly (since January 2017) by the General Practice Extraction Service of NHS Digital

Data release	Patients registered	Type 1 opt-outs		Type 2 opt-outs	
	(England)	*N*	%	*N*	%
April 2016	56 052 052	NA	NA	1 217,827	2.17
May 2016	56 257 680	1 436 377	2.55	1 228,832	2.18
June 2016	56 743 416	1 467 912	2.59	1 248 782	2.20
July 2016	57 111 251	1 494 565	2.62	1 263 738	2.21
August 2016	57 352 051	1 519 534	2.65	1 272 764	2.22
September 2016	57 492 142	1 534 950	2.67	1 279 950	2.23
October 2016	57 753 456	1 557 819	2.70	1 289 855	2.23
November 2016	57 842 512	1 583 597	2.74	1 303 062	2.25
December 2016	57 839 518	1 599 017	2.76	1 310 798	2.27
March 2017	58 056 288	1 652 059	2.85	1 332 090	2.29
June 2017	58 275 085	1 701 059	2.92	1 349 767	2.32
September 2017	58 444 055	1 746 976	2.99	1 362 672	2.33
December 2017	58 765 123	1 806 882	3.07	1 403 335	2.39

### Spatial patterns

**Fig. 2:**
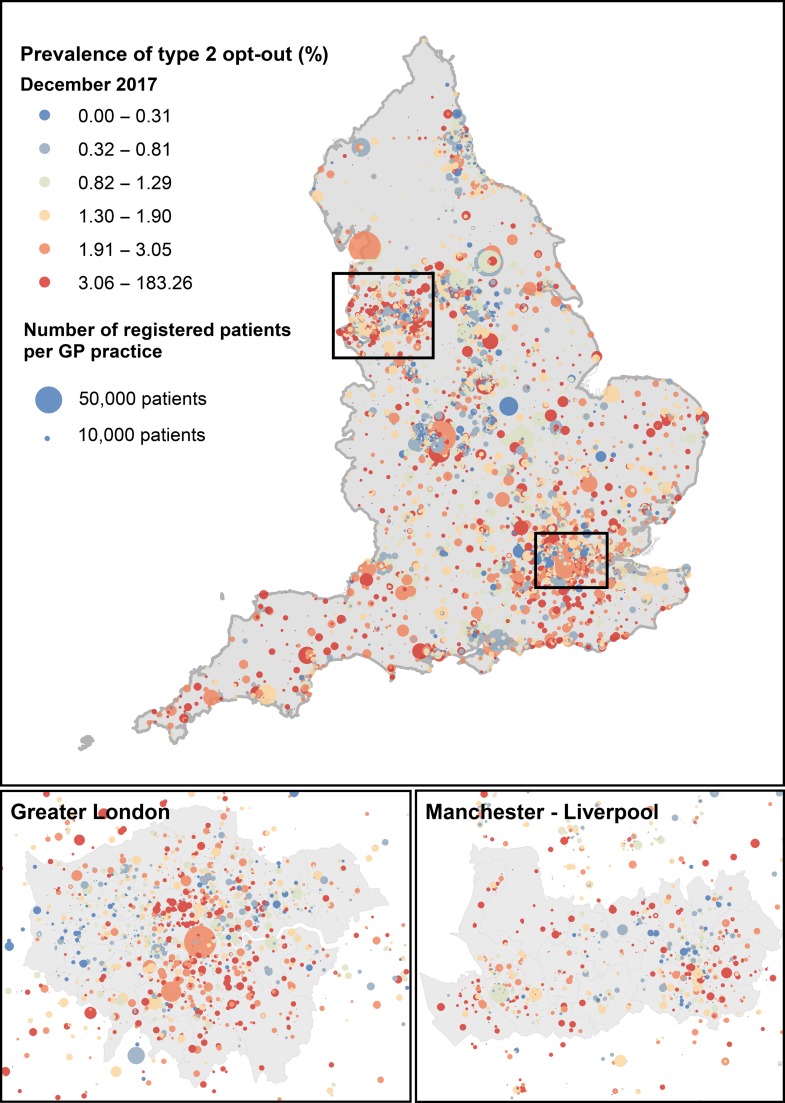
Map of the prevalence (%) of type 2 opt-outs in GP practices in England. Based on data released by NHS Digital in December 2017. Circles are proportional to the number of patients registered. The colour scale is based on quantiles.

There are striking differences in the geographical distribution of opt-outs at GP practice level. We linked data on opt-outs for December 2017 released by NHS Digital with postcodes from GP practices, also available from NHS Digital (https://digital.nhs.uk/organisation-data-service/data-downloads/gp-data) and geographical coordinates associated with postcode centroids, available from the Ordnance Survey Open Data (https://www.ordnancesurvey.co.uk/business-and-government/products/code-point-open.html). We then mapped the proportion of type 2 opt-outs using a geographical information system (GIS) (Fig. 2), allowing for a visual assessment of the spatial distribution of opt-outs. For example, the prevalence of type 2 opt-outs in Camden in North London was 9.06%, compared with 1.04% in Kensington and Chelsea in Central London and 2.3% on average in England.

**Fig. 3:**
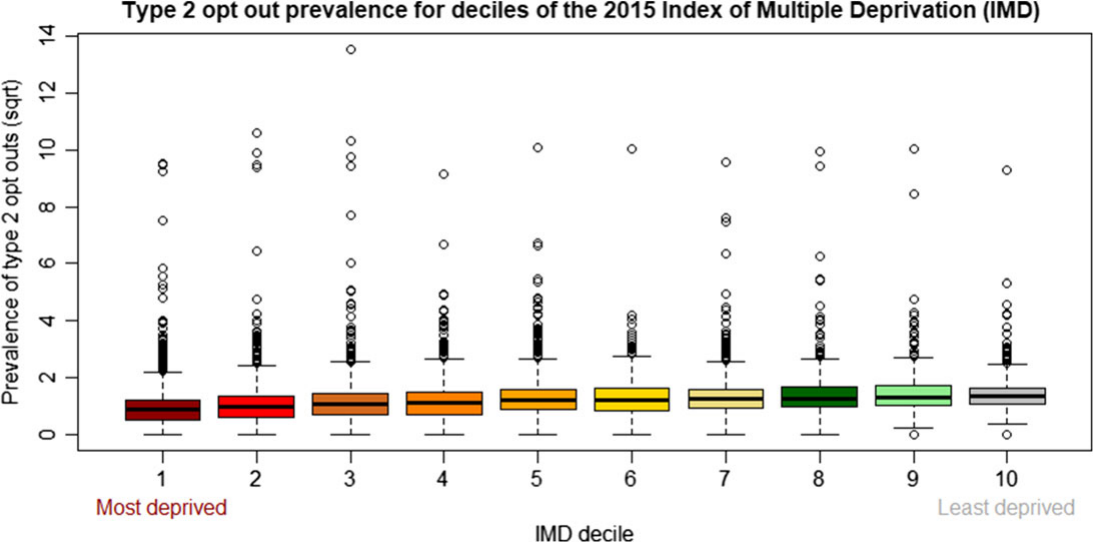
Boxplot of the prevalence of type 2 opt-outs at GP practice level according to deciles of the 2015 Index of Multiple Deprivation (IMD) at Lower Super Output Areas (LSOA) level. A square root (sqrt) transformation was applied to normalize the distribution of prevalence data.

Linking opt-out rates with 2015 Index of Multiple Deprivation,^17^ a measure of deprivation at census small-area level, i.e. Lower Super Output Areas (LSOA, available from the Open Data Communities (https://www.gov.uk/government/statistics/english-indices-of-deprivation-2015)) suggested a statistically significant association (ANOVA, *P* < 0.0001) between deprivation and opt-out rates, with a lower prevalence in the most deprived areas (first-fourth decile compared with 5th-10th decile) (Fig. 3).

### Small-area studies

**Fig. 4:**
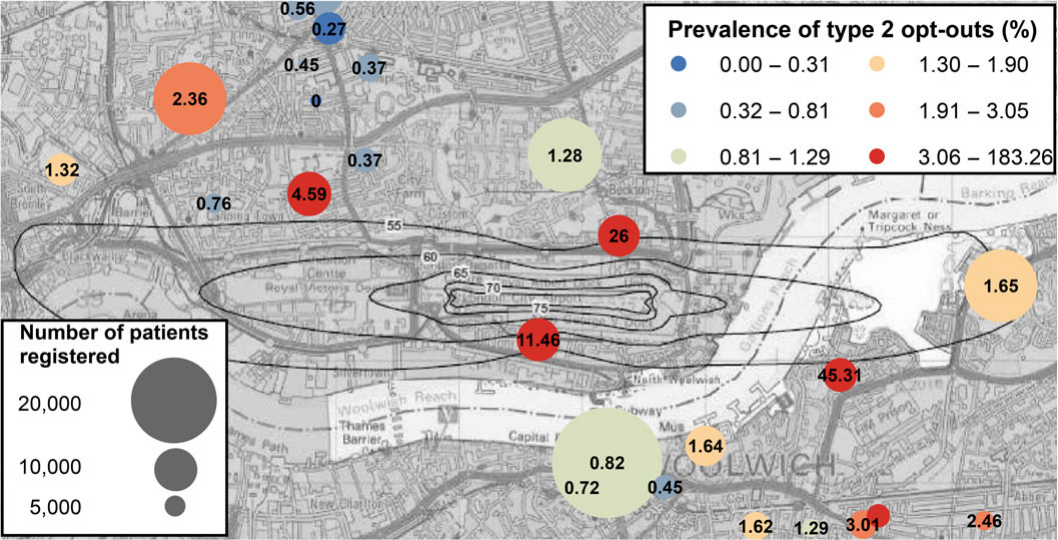
Prevalence of type 2 opt-outs in GP practices in the vicinity of London City Airport. The size of the circles is proportional to the number of patients registered in each GP practice. The colours indicate the prevalence of opt-outs based on NHS Digital data released in December 2017. The black line show noise contours with decibel (dB) levels as published in London City Airport’s Noise Action Plan 2013–18 (https://www.londoncityairport.com/content/pdf/Noise_Action_Plan_2013_2018.pdf).

Small-area studies are conducted at a local geographical level, such as individual addresses or postcodes (which comprise on average 18 households in England). The UK Small Area Health Statistics Unit (SAHSU, ww.sahsu.org) is a research unit set up in 1987 and now funded by Public Health England (PHE) as part of the national Medical Research Council (MRC)—PHE Centre for Environment and Health. Its remit is to carry out environmental health surveillance of the population in relation to risks to health of the population from industrial pollution and environmental contaminants with an emphasis on use and interpretation of routine health statistics (e.g. hospital admissions, mortality, birth and cancer registrations) at a small-area scale. National small-area studies conducted by SAHSU encompass a wide range of exposures, for example, surveillance of childhood cancers near nuclear installations; assessment of impacts of air pollution on birthweight;^18^ investigation of risks to foetal and infant health from waste incineration^19^ and to respiratory health from waste composting;^20^ cancer risks in relation to electromagnetic radiation exposure from mobile phone base stations^21^ and overhead powerlines.^22^ SAHSU also respond to requests by Public Health England to support their teams in rapidly investigating unusual clusters of disease, particularly in the neighbourhood of industrial installations. By nature, such analyses require access to sensitive health data at the individual level, with appropriate governance and security controls, for example, to link health records with local exposure to pollutants. High rates of opt-outs in specific geographic areas or socio-demographic groups could bias such investigations and potentially mask important associations. To illustrate this point, Fig. 4 shows the prevalence of opt-outs in GP practices surrounding London City airport. There are growing concerns about the health impact of aircraft noise and air pollution around airports.^23^ A recent SAHSU study of populations living near Heathrow before the opt-out system was introduced suggested higher risks of stroke and coronary heart disease in neighbourhoods exposed to high levels of aircraft noise.^24^ However, these neighbourhoods also had higher percentages of populations of non-white ethnicity. Given that opt-outs vary spatially and by ethnicity, this may introduce bias into similar studies in the future, and potentially result in false or misleading conclusions.

### Why does this matter?

A wide range of public health research studies, including surveillance and environmental health projects, could be particularly affected. Because of the high resolution used in small-area studies, these concerns are particularly relevant. Opt-outs introduce biases because they are not randomly distributed in space and time or by age, sex, ethnicity and socio-economic status. These biases might affect the accuracy and generalizability of results and therefore recommendations to translate research outcomes into public health policies. More explicitly, this could affect epidemiological studies by erroneously drawing attention to data gaps rather than real areas of interest, or by limiting the possibilities to infer information from neighbouring areas (e.g. to perform a Bayesian smoothing analysis^25^). Data gaps could also impede the detection of disease clusters with routine surveillance methods (such as BaySTDetect^26^). Furthermore, the opt-outs could bias the results of analysis of spatially varying environmental health hazards (e.g. incinerator and industrial emissions; bioaerosols from waste composting; environmental air pollution and transport noise exposure; electromagnetic fields from overhead power lines) in relation to non-communicable diseases. Finally, a careful assessment of the potential impact of opt-outs would likely slow down urgent investigations of short-term risks to environmental health using routine health data, and therefore increase the reaction time to take adequate measures to protect the public.

## Conclusions

In an increasingly digital world, offering enormous potential for big data analyses and precision medicine, many countries face the challenge of combining the collection of huge volumes of confidential and sensitive health records and reassuring the public about how these data are used. The NHS is one of the first public health institutions to try to tackle this balancing act between the control of data within a legal framework, the release of data to improve health and social care, patients’ right to determine how their health data are used, and the public’s trust.

While we support the option for patients to protect their health records from being used for purposes other than their direct care, with selected exemptions to protect public health, we sincerely believe that the lack of consideration of non-communicable diseases (and reproductive effects) in these exemptions could potentially have dramatic consequences for the ability to carry out public health research and surveillance of the population of England. Non-communicable (chronic) diseases represent a growing public health burden and their prevention and management is already becoming a considerable challenge for the NHS. A recent study conducted by PHE in collaboration with the Global Burden of Disease (GBD) Study^27^ showed that there are substantial opportunities for reductions in the burden of preventable disease, and concluded that systematic actions, locally and nationally, were needed to reduce risk exposures, support healthy behaviours, alleviate the severity of chronic disabling disorders, and mitigate the effects of socio-economic deprivation, all of which require access to the best available and most complete health data.

The Department of Health has recently announced that the new National Data Opt-out Programme will be introduced from March 2018, but many of the issues highlighted above will remain. We believe that failing to provide adequate exemptions for the study of non-communicable diseases for public health purposes within academic and public health institutions, where appropriate data security and governance systems are in place, risks substantially impeding these goals.
